# Lost in a Thyroid Storm – Psychosis as a First Presentation of Grave’s Disease

**DOI:** 10.1192/bjo.2025.10737

**Published:** 2025-06-20

**Authors:** Eneida Karriqi, Emma Padfield

**Affiliations:** 1 North East London NHS Foundation Trust, London, United Kingdom; 2 Oxleas NHS Foundation Trust, London, United Kingdom

## Abstract

**Aims:** Thyrotoxicosis is caused by an overactive thyroid gland, leading to excessive production and release of thyroid hormones into the bloodstream. The most common cause is Grave’s disease. Presentation of Grave’s disease with neurological and psychiatric symptoms as first line is rare; however it can lead to mis-diagnosis of a primary psychiatric condition, especially in younger patients. This report illustrates the case of a 24-year-old female presenting with psychotic symptoms on a background of undiagnosed Grave’s disease.

**Methods:** Case report.

A 24-year-old lady was brought to A&E due to an abrupt change in behaviour, with confusion, bizarre speech, attempts to run on the street, aggression and insomnia. At assessment, she presented with thought disorder, thought block and derealisation phenomena. She was physically well, with only some mild diarrhoea. Routine blood tests were done at initial presentation, but this did not include thyroid function tests. She was detained under the Mental Health Act and transferred to a psychiatric ward. Thyroid function tests revealed an extremely high thyroid hormone level and presence of thyroid receptor antibodies. She was started on carbimazole and propranolol and her psychotic symptoms improved markedly without antipsychotic medication. In the next few months, however, her psychiatric symptoms returned and she required further treatment in hospital as well as commencement of risperidone, an antipsychotic. There was much debate between psychiatry and endocrine teams about the appropriate place of her care and the legal framework for a young woman who lacked capacity to consent to treatment due to an organic psychosis.

**Results:** Albeit rarely, hyperthyroidism can present with acute onset disorientation which can be misdiagnosed as a primary psychiatric disorder. Prompt treatment of hyperthyroidism with antithyroid medications is crucial for mitigating psychiatric symptoms, but it may take several weeks to months for thyroid hormones to return to baseline. The use of antipsychotics should be considered for symptom management; the dose and duration of treatment will depend on the time needed for return to euthyroid state, severity of symptoms and persistence of psychotic symptoms after correction of thyroid balance. Close collaboration between psychiatrists and endocrinologists is essential for the patient to receive the best quality care. Involving the patient and their family in care is equally important to support recovery in the longer term.

**Conclusion:** This case highlights the importance of considering organic causes in patients presenting with psychiatric symptoms.

